# Macronutrient Composition of the Australian Population’s Diet; Trends from Three National Nutrition Surveys 1983, 1995 and 2012

**DOI:** 10.3390/nu10081045

**Published:** 2018-08-08

**Authors:** Amanda Grech, Anna Rangan, Margaret Allman-Farinelli

**Affiliations:** Nutrition and Dietetics Discipline, School of Life and Environmental Sciences, Charles Perkins Centre, The University of Sydney, Sydney 2006, Australia; anna.rangan@sydney.edu.au (A.R.); margaret.allman-farinelli@sydney.edu.au (M.A.-F.)

**Keywords:** overweight, macronutrients, dietary energy, sugar-sweetened beverages, dietary carbohydrate, dietary fat, dietary protein

## Abstract

Although the role of individual macronutrients in the development of obesity remains controversial, changes in macronutrient composition of the diet may have played a causal role in the obesity epidemic. The aim of this analysis was to determine the percentage energy (%E) for protein, carbohydrate and fat of Australian adults’ diets over time. Cross-sectional, national nutrition surveys from 1983, 1995 and 2012 assessed diet using one 24 h recall. The prevalence of obesity increased between each survey, from 9.6% to 19.7% and 27.7%. Protein (%E) differed between each survey and contributed 17.7%, 16.8% and 18.3% energy in 1983, 1995 and 2012, respectively (*p* < 0.001). Carbohydrate (%E) increased from 40.0% in 1983 to 44.9% in 1995 (*p* < 0.001), with no change in dietary fibre but declined in 2012 to 43.1%. Fat (%E) declined between each survey from 35.3%, 31.9%, to 30.9%, respectively (*p* < 0.001). Alcohol (%E) has declined for younger adults and men but intake increased for women aged >45 years. Prospective cohort studies with comprehensive assessment of foods consumed, together with measurements of weight and height, will advance the understanding of the relationship between macronutrients and changes in body weight and obesity.

## 1. Introduction

In 2015, it was estimated that globally 603.7 million adults (12%) were obese [1]. Overweight and obesity is associated with over 20 disease conditions including cardiovascular disease, some cancers and osteoarthritis and contributed 7.2% of mortality in 2015 [1]. Despite concerted attempts to stop the progression of the obesity epidemic, increases in the prevalence of obesity in adults have been witnessed and it is predicted obesity will continue to rise in the future without effective intervention [2]. Determining effective solutions to prevent further increases is paramount to resolving the obesity epidemic and ensuring the health of the population.

The causes of obesity are multi-factorial and complex, but broadly, weight gain is caused by energy imbalance when energy expenditure is insufficient to match dietary energy intake [3]. Increases in incident obesity have been attributed to reductions in physical activity and changes in dietary patterns [4,5]. There have been observed widespread changes in the food supply that coincide with the obesity epidemic.

Dietary energy is primarily provided by macronutrients; fat, carbohydrate and protein but also alcohol and dietary fibre [6]. The effect of fat, carbohydrate and protein on body weight has remained controversial. Since the 1990s research focused on reducing fat intake because it is the most energy-dense macronutrient, providing the most energy per gram [7]. While there is evidence that decreasing fat intake can lead to modest spontaneous weight loss [7], studies on the long-term benefits of a lower fat diet on body weight are inconsistent [8]. More recent research has focused on the role of carbohydrates in obesity [7]. Carbohydrates’ effect on body weight appears to be differential depending on the source of the carbohydrate [7]. In particular, sugar-sweetened beverages [9,10] and refined carbohydrates have been implicated in obesity [11,12], whereas consumption of other rich food sources of carbohydrates that are higher in dietary fibre such as wholegrains, are associated with lower body fatness [11,12,13].

The role of protein in the obesity epidemic has received relatively little attention [7]. Protein may be important in appetite regulation and in isogenic pre-loads of macronutrients in laboratory studies, protein leads to lower subsequent energy intake compared to other macronutrients [14]. The protein leverage hypothesis proposes that protein is the most tightly regulated macronutrient in adults [15]. The requirement for a minimum amount of dietary protein drives overall food intake to ensure that these requirements are met [15]. As a consequence, if protein requirements have not been met before energy requirements, more food will be consumed to ensure adequate protein intake, leading to energy imbalance and weight gain [15]. Evidence for the protein leverage hypothesis has largely been derived from animal model studies and evidence is needed for humans [16,17].

Current dietary recommendations for macronutrients have been established to meet nutrient requirements, while minimizing risk of adverse health. In Australia and New Zealand the acceptable macronutrient distribution range (AMDR) is 15–25% of energy from protein to ensure adequate intake of protein and other micronutrients, 20–35% from fat to sustain body weight and ensure adequate micronutrient intake and 45–65% from carbohydrate, to ensure adequate fat and protein intake [18]. The recommendation for carbohydrate stipulates that the source of carbohydrate should predominantly be from low glycemic index and low energy dense sources as the quality of carbohydrate is paramount to health [18]. It is recommended that alcohol contributes to less than 5% energy to allow adequate intake of nutrients and avoid weight gain [19]. These recommendations are consistent with those of the Food and Nutrition Board: Institute of Medicine, with the exception for protein which ranges from 10%, to meet minimum requirements for protein, to 35%, to accommodate carbohydrate and fat requirements [18].

Evidence from national nutrition surveys indicate that the percentage energy from protein and fat has decreased, while carbohydrate and total energy increased between 1971 and 2006 in the USA [20]. There has been no published assessment of the change of the contribution of macronutrients to nutrient intake over time in a representative sample of the Australian population.

The aim of the present analysis is to determine if the macronutrient composition of the diet has changed between 1983 and 2012.

## 2. Materials and Methods

### 2.1. Data Sources

The present analysis consists of secondary analysis of data collected in three cross-sectional Australian national nutrition surveys: the National Dietary Survey of Adults 1983 (NDSA-1983); the National Nutrition Survey, 1995 (NNS-1995); and the National Nutrition and Physical Activity Survey, 2011/12 (NNPAS-2011/12). The NNS-1995 and NNPAS-2011/12 were both conducted by the Australian Bureau of Statistics (ABS). The NDSA-1983 was conducted by the Heart Foundation in conjunction with the Commonwealth Department of Health as part of the Risk Factor Prevalence Study, 1983. Detailed methodologies for the three surveys are published elsewhere [21,22,23,24].

#### 2.1.1. NDSA-1983

The NSDA-1983 was a multi-staged area quota sample from the electoral role. Participants were selected from the electoral role from six Australian state capital cities within a 16 km radius of the National Heart Foundation centers. Eligible participants included adults aged 25–64 years. The interviews were scheduled on Monday–Friday and participants were asked to recall food intake for the previous 24 h (i.e., food intake was reported for Sunday through to Thursday). The survey was conducted from May to November 1983.

#### 2.1.2. NNS-1995 and NNPAS-2011/12

Both NNS-1995 and NNPAS-2011/12 surveys were stratified multi-staged area samples of private dwellings selected from across Australia. Eligible participants for the surveys included persons aged 2 years and over. Adults aged 18 years and over were included in the present analysis. Interviews were conducted Monday through to Sunday. The NNS-1995 was conducted from February 1995 to March 1996, and the NNPAS-2011/12 was conducted from May 2011 to June 2012.

#### 2.1.3. Outcomes

The outcomes of interest included the prevalence of obesity, total dietary energy and energy from macronutrients, expressed as percentage of total dietary energy from protein, fat, carbohydrate, dietary fibre and alcohol for subjects from the NDSA-1983, NNS-1995 and NNPAS-2011/12.

Weight (kg) and height (cm) were measured in each of the surveys by the interviewers. Body mass index (BMI) was calculated for each participant (weight kg/height m^2^). BMI was categorised in accordance with the World Health Organizations (WHO) definition as underweight (<18.5 kg/m^2^); normal (≥18.5 to <25.0 kg/m^2^); overweight (≥25.0 kg/m^2^ to <30.0 kg/m^2^) and obese (≥30.0 kg/m^2^). The percentage of participants in each weight category was calculated, excluding participants that did not have measurements taken.

Total dietary energy in the food composition databases used for the NDSA-1983 and NNS-1995 did not include dietary fibre; therefore, energy from dietary fibre was added to the total energy for each participant. In this way, total energy was consistent in all three surveys. Energy derived from each macronutrient was calculated by multiplying grams of each macronutrient by the appropriate Atwater factor: Carbohydrates = (sugars × 16 + starch × 17 + other available carbohydrates × 17 (i.e., glycogen + raffinose + stachyose + dextrins + maltodextrins + other undifferentiated oligosaccharides); fat × 37; protein × 17; alcohol × 29; dietary fibre × 8. Atwater factors used in the nutrient composition databases were obtained to remain consistent with other published nutrient values in Australia.

### 2.2. Dietary Assessment

All three surveys collected dietary data with 24 h recalls. The NDSA-1983 and the NNS-1995 interviews were pen and paper, personal interviews conducted by trained nutritionists and the NNPAS-2011/12 were computer-assisted personal interviews (CAPI) conducted by trained and experienced personnel selected from the ABS panel. Each survey used standardized 24 h recall protocols. The NDSA-1983 protocol involved a detailed interview, using a pre-determined set of probing questions, which took an average 25 min to complete [21]. Although no further details are available for the 1983 protocol, the methodologies of the three surveys are expected to produce comparable estimates of food and nutrient intakes [21,25].The 3-pass and 5-pass method used for the NNS-1995 and NNPAS-2011/12 surveys respectively, were originally developed by the United States Department of Agriculture (USDA) and were modified to reflect the Australian food supply by the government agency, Food Standards Australia Zealand (FSANZ) (formally known as Australia New Zealand Food Authority (ANZFA)) in conjunction with the USDA. A second recall was collected for a subset of the NNS-1995 and NNPAS-2011/12 survey population; however, a second recall was not collected for the NDSA-1983 population. Data from the second interview was therefore not included in the present analysis.

Three nutrient composition databases constructed by FSANZ were used to derive nutrient composition. The original nutrient composition database developed for NDSA-1983 predominantly obtained food composition from international sources and therefore was not representative of the Australian food supply. To address this, an updated database was created by the ANZFA in 1991 to reflect the Australian food supply and the original food composition database was replaced with the new database. Codes to match the foods in each database were supplied in the bridging study [21]. The food composition databases AUSNUT-1999 and AUSNUT-2013 were each specifically developed for the NNS-1995 and the NNPAS-2011/12 surveys, respectively.

### 2.3. Energy Misreporting

Participants that reported implausible, low energy intake were identified with the Goldberg equation, as those with a reported energy intake to basal metabolic rate ratio (EI: BMR) of <0.87 [26]. Analysis was conducted to assess the number of participants in each survey with implausibly low energy intakes and all analyses described below were conducted with and without participants to assess the impact of energy misreporting on the trends in macronutrients reported here. Analyses excluding those identified as misreporters are presented as supplementary material (Supplementary Table S1). Removing misreporters had no material effect on the percentage energy derived from macronutrients, and therefore the data presented in the text includes all participants.

### 2.4. Sensitivity Analysis

There were several methodological differences for the NDSA-1983, compared to the NNS-1995 and NNPAS-2011/12 that had comparable methods to each other. Differences between the surveys included: (1) Age of the participants, (2) Geographic areas the participants lived in, (3) Seasons of the data collection, (4) Days of the week on which foods consumed were reported. To find an estimate of nutrient intakes for the 1995 and 2011/12 surveys that were not different simply due to sampling differences between surveys, the mean differences in macronutrients (%E) for age groups (18–24, 25–34, 35–44, 45–54, 55–64 and 65 years and over); geographic areas, (Metropolitan and all other areas); months (December–April and May–November) and days of the week foods consumed (Sunday–Thursday and Friday and Saturday) were calculated. Supplementary Table S2 presents the estimates in macronutrients where only participants in the NNS-1995 and NNPAS-2012 were comparable to NDSA-1983 survey population were included. Age and weekend alcohol intake significantly affected estimates and as subjects aged <25 and >64 years and weekend diet (i.e., Friday and Saturday intake) was not included in NDSA-1983. As such for comparisons between the three surveys, only subjects aged 25–64 years were compared and alcohol estimates were calculated excluding participants that reported intake on the weekend to ensure that estimates are comparable.

### 2.5. Statistical Analysis

Differences between surveys (1983, 1995 and 2012) in energy from macronutrients (%E) (protein, carbohydrate, fat, alcohol and fibre) were assessed for men, women and the total population and for age groups (18–24, 25–44, 45–64 and 65 years and over). Analysis of variance and *t*-tests were used to assess mean differences and Tukey’s post hoc test was used to determine group differences. *p*-values < 0.001 were considered statistically significant to minimize the chance of Type I error. Sampling weights supplied with the datasets were applied to make the sample representative of the Australian population at the time of the survey. Analyses was conducted using SAS software (Version 9.4 for Windows © 2002–2012 SAS Institute Inc., Cary, NC, USA).

## 3. Results

### 3.1. Changes in Body Mass Index

The proportion of participants categorised as underweight, normal, overweight and obese is shown in Table 1. The prevalence of participants with a BMI > 25.0 kg/m^2^ increased in each survey, as did the prevalence of obesity, which increased between each survey and was 9.6% in 1983, 19.7% 1995 and 27.7% in 2012. Males and females demonstrated similar prevalence of obesity at each year surveyed. Increases in obesity were seen for all age groups for both males and females between each survey.

### 3.2. Change in Energy and Macronutrient Intake between 1982, 1995 and 2012

Mean (±95 Confidence Interval) energy intake for participants aged 25–64 years was 9079 kJ (8985–9174) in 1983, increased to 9389 kJ (9301–9478) in 1995 and declined to 8627 kJ (8538–8715) in 2012. The highest energy intake was seen in 1995 for both men and women and for men in 1983, 1995 and 2012 was 11,047 kJ (10,907–11,187), 11,147 kJ (11,012–11,281), 9903 (9765–10,040), respectively and for women was 7317 kJ (7224–7411), 7614 kJ (7526–7703), and 7372 kJ (7275–7468), respectively.

The percentage of dietary energy obtained from macronutrients protein, carbohydrates, fat, alcohol and dietary fibre are shown for men, women and the total population for each survey in Table 2. There was a small but significant decrease in protein (%E) between 1983 and 1995 from 17.7% to 16.8% and then an increase again to the highest estimate by 2012, at 18.3% (*p* < 0.001). This trend was seen for both men and women (Table 2). Protein contributed more energy for those in older age groups up to age 65 years (Table 3 and Table 4).

Carbohydrate contributed the most energy of the macronutrients (Table 2). Energy from carbohydrates increased between 1983 and 1995 from 40.0% to 44.9% respectively, and then declined between 1995 and 2011/12 to 43.1% (Table 2). Carbohydrate intake declined with increasing age and in NNPAS-2011/12 contributed 45.6% of energy for young adults aged 18–24 years compared to 42.8% for those aged 65 years and over. Energy from fat declined between each survey from 35.3% in 1983 to 30.9% in 2012. There was a small difference in fat intake for each age group, decreasing with age (Table 3 and Table 4).

There was no change in dietary fibre intake between 1983 and 1995 contributing 2% of energy in both years, but a small and significant increase was noted between 1995 and 2012 for men but not women (Table 2). Dietary fibre intake increased with age in each survey and was higher for women compared to men.

The percentage energy contribution from alcohol decreased between 1983 from 4.7% to 3.7% in 1995, with a small but significant increase between 1995 and 2012 to 4.3%. These estimates slightly under-estimate alcohol (%E), due to the comparable sample excluding weekend intake. When participants from the full sample aged 18 years and over in the NNS-1995 and NNPAS-2012 were included, alcohol contributed 3.6%E in 1995 and 4.3%E in 2012 (Supplementary Table S2). The increase was due to increased intake for women aged >45 years from 2.5 to 4.2%E. For all other age groups there was no significant changes between 1995 and 2012. With and without weekend data, alcohol (%E) increased with age, peaking at age 50–64 in each survey and declined after age 65. Men had higher alcohol (%E) than women in each survey.

## 4. Discussion

In Australia, the prevalence of obesity has increased from 10% in 1983, to 20% in 1995, and 28% in 2012. During this time significant changes in the energy contribution of macronutrients have been observed; between 1983 and 1995, despite reductions in intake of fat, protein and alcohol, energy intake increased, together with carbohydrate. The increase in carbohydrate was not accompanied with an increase in dietary fibre. In contrast, between 1995 and 2012, when the prevalence of obesity had slowed, there was a small but significant reduction in both carbohydrate and fat intake, an increase in dietary fibre and protein for all subgroups and alcohol overall. These data suggest a substantial change in the population’s diet during the peak of the obesity epidemic and some indications of improvement between 1995 and 2012.

There have been surprisingly few population surveys that have tracked macronutrient intake by measuring diet for an extended period. However, surveys that have done so have shown similar trends to those reported here. In the United States (USA) between 1971 and 2006 and in Germany between 1984 and 2000, protein (%E) and fat decreased in the USA, while carbohydrate intake increased in both the USA and Germany [20,27,28].

Diluting the protein content of the diet, as was seen between 1983 and 1995, may also have played a role in overconsumption. The protein leverage hypothesis states that there are inherent protein requirements that drive food intake [15]. It is possible that increased intake of foods with a lower protein content would require greater compensatory food intake to meet protein requirements [29]. The obesity epidemic correlates with observed increases in the availability of energy dense, nutrient poor foods that are high in added fat and sugars [5] but also low in protein. If the protein leverage hypothesis is correct, then greater intake of these foods may contribute to overconsumption because they do not provide adequate satiety [15].

There are a number of proposed mechanisms that the change in carbohydrate could have on body weight. Dietary fibre and resistant starch, have been associated with lower adiposity in randomized controlled trials [13] and epidemiological studies [12,30]. Dietary fibre increases chewing time and gastric distention and delays gastric emptying improving feelings of fullness and alters the postprandial glucose and insulin responses, which have been associated with greater satiation [7,31]. Furthermore, emerging evidence suggests that dietary fibre encourages growth of a more favorable gut microbiome, associated with lower long-term weight gain [32] and increased ease of weight loss [31]. It has been reported that the glycemic index (GI) and glycemic load (GL) of the diet also improved between 1995 and 2012, decreasing by 5% and 12%, respectively. Diets with a lower GI have been proposed to protect against obesity [31].

Fat has been regarded as potentially ‘obesogenic’ in the past because it is the most energy-dense macronutrient providing 37 kJ per gram as opposed to carbohydrate and protein which provide ~17 kJ per gram [33] and has been shown to be weakly satiating [31]. Interestingly, despite decreases in fat, there has been an increase in the dietary energy density of the Australian and US populations’ diets over this time period [34,35]. This is possible as energy density is not only determined by fat content of foods but also the moisture and dietary fibre content, for example, dry carbohydrate foods such as confectionary have a relatively high energy density [36]. As dietary energy density has not been reduced with a reduction of fat intake, this may explain why the obesity epidemic occurred concurrently with a reduced fat intake.

Despite increases in carbohydrate, 51% of Australians fail to meet the AMDR for carbohydrate in 2012 [37], and are also failing to consume predominantly lower energy dense, low glycemic sources of carbohydrate [38]. Recommendations should therefore emphasize the importance of replacing current carbohydrates with higher dietary fibre, low-GI options rather than emphasizing reducing carbohydrate intake [38]. Furthermore, 13% of men and 16% of women consumed fat intake above the AMDR despite reductions in fat intake between surveys, while 11% of men and 13% of women had intakes below the AMDR for protein [37].

Alcohol has also made a significant contribution to energy in Australia, particularly for males, and in 2012 more than 50% of males and 30% of females aged >50 exceeded the National Health and Medical Research Council (NHMRC) recommendation of 5% [19]. Changes in alcohol intake between surveys varied for different groups and the contribution to energy has decreased for younger adults while it has increased or remained stable for older adults. This is consistent with other research in Australia, with decreases shown for young adults, while increased intakes have been observed for middle aged women [39,40]. Alcohol appears to be additive to dietary energy intake from food and may contribute to weight gain, although the epidemiological evidence has varied [41].

### Limitations

The analyses use cross-sectional data from three separate surveys rather than a true cohort and therefore it is not possible to demonstrate causation. Self-reported dietary data is known to have limitations including misreporting of energy intake. However, removing participants that reported implausible energy intakes did not change the reported macronutrient trends. Evidence from validation studies of 24-h recalls using unbiased biomarkers of energy and protein intakes have demonstrated that the use of nutrient densities, such as percentage energy used here, can somewhat address this issue [42]. While energy was underestimated by approximately 16–20% in women and 12–14% in men, protein was relatively more accurately reported with underestimation of 11–12% for men and 11–15% for women [42]. However, protein expressed as percentage of energy was slightly overestimated and for men contributed 17.1% of energy as measured with the biomarker compared to 17.8–18.3% with 24 h recalls, and for women 16.1 - 16.3% compared to 17.4% with the 24 h recall [43]. Therefore, it is expected that protein (%E) is overestimated here and to a greater extent in the NNPAS-2011/12 for men where the proportion of low-energy reporting increased, while other macronutrients are likely to be underestimated. The error in the estimated protein (%E) was in the range of 0.7–1.3% [43], which is within the range of the change that we have seen here. In addition, it was not possible to determine how or when macronutrients changed over the 17-year period between 1995 and 2012, as there were no nationally representative surveys conducted during this period. Some differences in the ways that foods were classified in the 1983 survey compared with the 1995 and 2011/12 surveys has meant that while nutrients can be compared, the comparison of the foods themselves across the three surveys was not possible.

## 5. Conclusions

There have been changes in the macronutrient composition of the diet between 1983 and 2012. Australians reported consuming less protein, alcohol and fat but a considerable increase in carbohydrate at the time when there was a doubling of the prevalence of obesity between 1983 and 1995. Since 1995, rates of obesity have slowed and this has coincided with an increase in protein, fibre and alcohol and a small but significant decreases in both fat and carbohydrate. There is need for continued surveillance of the populations’ diet and obesity prevalence. Consideration must be given to the food classification systems used in future surveys so direct comparisons can be made. Prospective cohort studies with comprehensive assessment of foods consumed, together with measurements of weight and height, will advance the understanding of the relationship between macronutrients and changes in body weight and obesity.

## Figures and Tables

**Table 1:** Prevalence of overweight and obesity of Australian men and women aged 25–64 years in national nutrition surveys from 1983, 1995 and 2012.

			Body Mass Index Category			
Sex	Year	(*n*)	Underweight	Normal	Overweight	Obese
**Males**	**1983**	3021	1.2	48.9	40.5	9.4
	**1995**	3660	0.3	31.6	48.2	19.8
	**2012**	2683	0.8	27.0	44.5	27.8
**Females**	**1983**	3233	3.6	65.7	21.0	9.8
	**1995**	3958	1.8	48.4	30.3	19.5
	**2012**	2865	1.8	40.8	29.9	27.6
**Total Population**	**1983**	6254	2.5	57.8	30.2	9.6
	**1995**	7618	1.1	39.8	39.5	19.7
	**2012**	5548	1.3	33.7	37.4	27.7

Survey weights applied. Underweight (<18.5 kg/m^2^); normal (18.5 to <25.0 kg/m^2^); overweight (25.0 kg/m^2^ to <30.0 kg/m^2^) and obese (≥30.0 kg/m^2^).

**Table 2:** Change in percentage energy (%E) of macronutrients for Australian adults aged 25–64 years from National Nutrition Surveys 1983 (*n* = 6254), 1995 (*n* = 7831) and 2012 (*n* = 6552).

Macronutrient	Survey	Men		Women		Total Population	
		Mean	(± 95% CI)	Mean	(± 95% CI)	Mean	(± 95% CI)
Protein	1983	17.4 ^a^	(17.2–17.5)	18.0 ^a^	(17.8–18.2)	17.7 ^a^	(17.6–17.8)
	1995	16.8 ^b^	(16.6–16.9)	16.9 ^b^	(16.7–17.0)	16.8 ^b^	(16.7–16.9)
	2012	18.2 ^c^	(18.0–18.4)	18.5 ^c^	(18.3–18.7)	18.3 ^c^	(18.2–18.5)
	*p*-Value	<0.0001		<0.0001		<0.0001	
Carbohydrate	1983	39.1 ^a^	(38.8–39.5)	40.8 ^a^	(40.5–41.1)	40.0 ^a^	(39.8–40.3)
	1995	44.1 ^b^	(43.7–44.4)	45.8 ^b^	(45.5–46.1)	44.9 ^b^	(44.7–45.1)
	2012	43.0 ^b^	(42.6–43.4)	43.1 ^c^	(42.7–43.5)	43.1 ^c^	(42.8–43.3)
	*p*-Value	<0.0001		<0.0001		<0.0001	
Fat	1983	35.2 ^a^	(34.9–35.5)	35.3 ^a^	(35.0–35.6)	35.3^a^	(35.0–35.5)
	1995	31.9 ^b^	(31.6–32.1)	31.9 ^b^	(31.6–32.2)	31.9 ^b^	(31.7–32.1)
	2012	30.5 ^c^	(30.2–30.9)	31.3 ^c^	(31.0–31.6)	30.9 ^c^	(30.7–31.1)
	*p*-Value	<0.0001		<0.0001		<0.0001	
Alcohol ^‡^	1983	6.2 ^a^	(5.9–6.5)	3.4 ^a^	(3.1–3.6)	4.7 ^a^	(4.5–4.9)
	1995	4.7 ^b,c^	(4.4–5.0)	2.6 ^a^	(2.4–2.8)	3.7 ^b,c^	(3.5–3.8)
	2012	5.0 ^c^	(4.7–5.4)	3.5 ^a^	(3.2–3.8)	4.3 ^a,c^	(4.0–4.5)
	*p*-Value	0.0004		0.0604		0.0006	
Dietary Fibre	1983	1.9 ^a^	(1.8–1.9)	2.2 ^a^	(2.2–2.3)	2.1 ^a^	(2.0–2.1)
	1995	1.9 ^a^	(1.9–1.9)	2.2 ^a^	(2.2–2.3)	2.1 ^a^	(2.0–2.1)
	2012	2.1 ^b^	(2.0–2.1)	2.3 ^a^	(2.3–2.4)	2.2 ^b^	(2.2–2.2)
	*p*-Value	<0.0001		0.0704		<0.0001	

Survey weights applied. ^‡^ Estimate for alcohol in 1995 and 2012 excludes participants that reported alcohol Friday and Saturday to provide a comparable estimate to the 1983 survey population (1995 *n* = 6719; 2012 *n* = 5478). *p*-values indicate differences between surveys determined with ANOVA. Tukey’s post hoc significant differences between groups indicated with a different letter.

**Table 3:** Change in percentage energy (%E) from macronutrients for Australian men by age group between 1983 (*n* = 3021), 1995 (*n* = 5146) and 2012 (*n* = 4329) for men from three national nutrition surveys.

		Survey						
		1983		1995		2012		
Nutrient	Age	Mean	95% CI	Mean	95% CI	Mean	95% CI	*p*-Value
**Protein**	**18–24**			16.1	(15.8–16.5)	19.0	(18.3–19.7)	<0.0001
	**25–44**	17.1	(16.8–17.3)	16.5	(16.3–16.7)	18.2	(17.9–18.5)	<0.0001
	**45–64**	17.9	(17.7–18.2)	17.2	(16.9–17.4)	18.1	(17.8–18.5)	0.0476
	**65+**			16.6	(16.3–16.9)	18.2	(17.8–18.5)	<0.0001
**Carbohydrate**	**18–24**			46.7	(45.9–47.4)	45.4	(44.3–46.4)	0.0394
	**25–44**	39.6	(39.1–40.0)	44.4	(44.0–44.9)	43.7	(43.2–44.2)	<0.0001
	**45–64**	38.3	(37.8–38.8)	43.5	(43.0–44.0)	42.2	(41.6–42.8)	<0.0001
	**65+**			45.1	(44.5–45.7)	42.9	(42.2–43.7)	<0.0001
**Fat**	**18–24**			32.3	(31.6–32.9)	30.7	(29.9–31.6)	0.0037
	**25–44**	35.6	(35.2–36.0)	32.3	(31.9–32.6)	30.7	(30.3–31.2)	<0.0001
	**45–64**	34.6	(34.1–35.1)	31.3	(30.9–31.7)	30.3	(29.8–30.8)	<0.0001
	**65+**			30.9	(30.4–31.4)	29.7	(29.1–30.3)	0.004
**Alcohol** ^‡^	**18–24**			2.4	(1.9–3.0)	2.4	(1.7–3.1)	0.8902
	**25–44**	5.8	(5.3–6.2)	4.2	(3.9–4.6)	4.0	(3.6–4.4)	0.0015
	**45–64**	6.9	(6.4–7.4)	5.4	(4.9–5.8)	6.2	(5.6–6.7)	0.0454
	**65+**			4.6	(4.1–5.1)	5.7	(5.1–6.2)	0.0105
**Fibre**	**18–24**			1.6	(1.5–1.7)	1.8	(1.7–1.9)	<0.0001
	**25–44**	1.8	(1.8–1.9)	1.8	(1.8–1.8)	2.0	(2.0–2.0)	0.0021
	**45–64**	2.0	(1.9–2.0)	2.1	(2.0–2.1)	2.1	(2.1–2.2)	0.0063
	**65+**			2.3	(2.2–2.3)	2.4	(2.4–2.5)	0.0003

Survey weights applied. ^‡^ Estimate for alcohol in 1995 and 2012 excludes participants that reported alcohol Friday and Saturday to provide a comparable estimate to the 1983 survey population. *p*-values indicate differences between surveys determined with ANOVA.

**Table 4:** Change in percentage energy (%E) from macronutrients for Australian women by age group between 1983 (*n* = 3233), 1995 (*n* = 5840) and 2012 (*n* = 5106) for men from three national nutrition surveys.

		Survey						
		1983		1995		2012		
Nutrient	Age	Mean	95% CI	Mean	95% CI	Mean	95% CI	*p*-Value
**Protein**	**18–24**			15.8	(15.4–16.2)	17.6	(17.0–18.2)	<0.0001
	**25–44**	17.8	(17.5–18.0)	16.5	(16.3–16.6)	18.2	(17.9–18.4)	0.0002
	**45–64**	18.4	(18.1–18.7)	17.5	(17.3–17.7)	18.9	(18.6–19.2)	0.0142
	**65+**			17.1	(16.8–17.4)	19.0	(18.7–19.3)	<0.0001
**Carbohydrate**	**18–24**			48.0	(47.2–48.7)	45.7	(44.8–46.7)	0.0003
	**25–44**	40.8	(40.4–41.3)	46.0	(45.6–46.4)	44.2	(43.6–44.7)	<0.0001
	**45–64**	40.8	(40.3–41.3)	45.4	(44.9–45.9)	42.0	(41.4–42.5)	<0.0001
	**65+**			46.7	(46.1–47.2)	42.7	(42.1–43.2)	<0.0001
**Fat**	**18–24**			32.1	(31.5–32.7)	32.0	(31.1–32.9)	0.8506
	**25–44**	35.5	(35.1–35.9)	32.3	(32.0–32.7)	31.5	(31.1–31.9)	<0.0001
	**45–64**	35.0	(34.5–35.4)	31.3	(30.9–31.7)	31.0	(30.6–31.5)	<0.0001
	**65+**			31.3	(30.8–31.8)	31.0	(30.5–31.5)	0.4454
**Alcohol** ^‡^	**18–24**			1.6	(1.2–2.0)	1.8	(1.1–2.6)	0.5337
	**25–44**	3.5	(3.2–3.8)	2.5	(2.2–2.8)	2.6	(2.3–3.0)	0.0147
	**45–64**	3.2	(2.8–3.5)	2.8	(2.5–3.1)	4.4	(4.0–4.9)	0.0039
	**65+**			1.8	(1.5–2.1)	3.6	(3.1–4.0)	<0.0001
**Fibre**	**18–24**			1.8	(1.8–1.9)	2.1	(2.0–2.2)	<0.0001
	**25–44**	2.2	(2.1–2.2)	2.1	(2.0–2.1)	2.2	(2.2–2.3)	0.1866
	**45–64**	2.4	(2.3–2.4)	2.4	(2.4–2.5)	2.4	(2.4–2.5)	0.5135
	**65+**			2.6	(2.5–2.6)	2.7	(2.6–2.8)	0.1311

Survey weights applied. ^‡^ Estimate for alcohol in 1995 and 2012 excludes participants that reported alcohol Friday and Saturday to provide a comparable estimate to the 1983 survey population. *p*-values indicate differences between surveys determined with ANOVA.

## Supplementary Materials

The following are available online at http://www.mdpi.com/2072-6643/10/8/1045/s1, Table S1: Change in energy from macronutrients from Australian National Nutrition Surveys 1983 (*n* = 5062), 1995 (*n* = 6284) and 2012 (*n* = 4380) for plausible energy reporters. Table S2: Sensitivity analysis to assess the impact of sampling on percentage energy from macronutrients in the National Nutrition Survey, 1995 and the National Nutrition and Physical Activity Survey, 2011/12 to find a comparable sample to the National Dietary Survey of Adults, 1983.
