# Supplementary material for: Macronutrient Composition of the Australian Population’s Diet; Trends from Three National Nutrition Surveys 1983, 1995 and 2012
Source: Nutrients. 2018 Aug 8;10(8):1045. doi: 10.3390/nu10081045 (PMC6115811; doi:10.3390/nu10081045)
Supplement: Supplementary file 1 [file nutrients-10-01045-s001.pdf]

Supplementary Table S1. Change in percentage energy from macronutrients from Australian National Nutrition Surveys 1983 (n = 5062), 1995 (n = 6284) and 2012 (n = 4380) excluding low energy-reporters

		Men		Women		Total Population	
**Macronutrient**	**Survey**	**Mean**	**(±95% CI)**	**Mean**	**(±95% CI)**	**Mean**	**(±95% CI)**
**Protein**	1983	17.1	(16.9–17.3)	17.3	(17.1–17.5)	17.2	(17.1–17.3)
	1995	16.6	(16.4–16.8)	16.6	(16.4–16.7)	16.6	(16.5–16.7)
	2012	17.7	(17.5–18.0)	17.9	(17.7–18.1)	17.8	(17.7–18.0)
	P-Value	*0.0012*		*0.0006*		*<.0001*	
**Carbohydrate**	1983	39.0	(38.7–39.4)	40.6	(40.3–41.0)	39.8	(39.6–40.1)
	1995	44.0	(43.7–44.3)	45.0	(44.7–45.4)	44.5	(44.2–44.7)
	2012	42.6	(42.2–43.1)	42.3	(41.9–42.8)	42.5	(42.2–42.8)
	P-Value	*<.0001*		*<.0001*		*<.0001*	
**Fat**	1983	35.7	(35.4–36.0)	36.0	(35.7–36.3)	35.9	(35.6–36.1)
	1995	32.1	(31.8–32.4)	32.8	(32.5–33.1)	32.4	(32.2–32.6)
	2012	30.9	(30.5–31.3)	32.2	(31.8–32.6)	31.5	(31.3–31.8)
	P-Value	*<.0001*		*<.0001*		*<.0001*	
**Alcohol** ‡	1983	6.2	(5.8–6.5)	3.7	(3.4–3.9)	4.9	(4.7–5.1)
	1995	4.8	(4.5–5.1)	2.9	(2.6–3.1)	3.9	(3.7–4.1)
	2012	5.7	(5.3–6.2)	4.2	(3.8–4.5)	5.0	(4.7–5.3)
	P-Value	*0.0004*		*0.0604*		*0.0006*	
**Dietary Fibre**	1983	1.8	(1.8–1.9)	2.1	(2.1–2.1)	2.0	(1.9–2.0)
	1995	1.9	(1.9–1.9)	2.1	(2.1–2.1)	2.0	(2.0–2.0)
	2012	2.0	(1.9–2.0)	2.2	(2.2–2.3)	2.1	(2.1–2.1)
	P-Value	*0.0003*		*0.0533*		*0.0002*	

Low energy-reporters are defined as those with energy intake: basal metabolic rate ratio between <0.87

%E; percentage energy

Survey weights applied

P-value; mean differences determined with ANOVA

Supplementary Table S2: Sensitivity analysis to assess the impact of sampling on percentage energy from macronutrients in the National Nutrition Survey, 1995 and the National Nutrition and Physical Activity Survey, 2011/12 to find a comparable sample to the National Dietary Survey of Adults, 1983^

Macronutrient	Sex	Survey	Mean	95 % CI	P–Value
Protein	Males	1983	17.4	(17.2–17.5)	
		1995	16.9	(16.6–17.2)	
		2012	18.4	(18.1–18.7)	<.0001
	Females	1983	18.0	(17.8–18.2)	
		1995	16.9	(16.6–17.1)	
		2012	18.5	(18.2–18.8)	<.0001
	Total Population	1983	17.7	(17.6–17.8)	
		1995	16.9	(16.7–17.1)	
		2012	18.5	(18.2–18.7)	<.0001
Carbohydrate	Males	1983	39.1	(38.8–39.5)	
		1995	44.1	(43.5–44.7)	
		2012	43.5	(42.9–44.0)	<.0001
	Females	1983	40.8	(40.5–41.1)	
		1995	46.2	(45.7–46.8)	
		2012	43.5	(42.9–44.0)	<.0001
	Total Population	1983	40.0	(39.8–40.3)	
		1995	45.2	(44.7–45.6)	
		2012	43.5	(43.1–43.8)	<.0001
Fat	Males	1983	35.2	(34.9–35.5)	
		1995	32.0	(31.5–32.5)	
		2012	30.5	(30.1–31.0)	<.0001
	Females	1983	35.3	(35.0–35.6)	
		1995	31.8	(31.2–32.3)	
		2012	31.2	(30.8–31.6)	<.0001
	Total Population	1983	35.3	(35.0–35.5)	
		1995	31.9	(31.5–32.2)	
		2012	30.9	(30.6–31.2)	<.0001
Alcohol	Males	1983	6.2	(5.9–6.5)	
		1995	4.7	(4.2–5.2)	
		2012	4.7	(4.3–5.2)	0.0252
	Females	1983	3.4	(3.1–3.6)	
		1995	2.5	(2.1–2.8)	
		2012	3.4	(3.1–3.8)	0.3934
	Total Population	1983	4.7	(4.5–4.9)	
		1995	3.6	(3.3–3.9)	
		2012	4.1	(3.8–4.3)	<.0001
Fibre	Males	1983	1.9	(1.8–1.9)	
		1995	2.0	(1.9–2.0)	
		2012	2.1	(2.1–2.1)	0.001
	Females	1983	2.2	(2.2–2.2)	
		1995	2.3	(2.2–2.3)	
		2012	2.4	(2.4–2.4)	0.0713

Total Population	1983	2.1	(2.0–2.1)	
	1995	2.1	(2.1–2.1)	
	2012	2.3	(2.2–2.3)	0.0003

Survey weights applied

P–values; mean differences determined with ANOVA or t-tests

^ Estimates for each survey derived from participants aged 25–64 years, with reported intake on Sunday to Thursday, interview months May to November and from capital cities of Australia.
